# Effects of Feeding Bt Maize to Sows during Gestation and Lactation on Maternal and Offspring Immunity and Fate of Transgenic Material

**DOI:** 10.1371/journal.pone.0047851

**Published:** 2012-10-16

**Authors:** Stefan G. Buzoianu, Maria C. Walsh, Mary C. Rea, Orla O'Donovan, Eva Gelencsér, Gabriella Ujhelyi, Erika Szabó, Andras Nagy, R. Paul Ross, Gillian E. Gardiner, Peadar G. Lawlor

**Affiliations:** 1 Teagasc, Pig Development Department, Animal and Grassland Research and Innovation Centre, Moorepark, Fermoy, Co. Cork, Ireland; 2 Department of Chemical and Life Sciences, Waterford Institute of Technology, Waterford, Co. Waterford, Ireland; 3 Teagasc Food Research Centre, Moorepark, Fermoy, Co. Cork, Ireland; 4 Alimentary Pharmabiotic Centre, University College Cork, Cork, Co. Cork, Ireland; 5 Central Food Research Institute, Budapest, Hungary; Universidad Nacional Autonoma de Mexico, Instituto de Biotecnologia, Mexico

## Abstract

**Background:**

We aimed to determine the effect of feeding transgenic maize to sows during gestation and lactation on maternal and offspring immunity and to assess the fate of transgenic material.

**Methodology/Principal Findings:**

On the day of insemination, sows were assigned to one of two treatments (n = 12/treatment); 1) non-Bt control maize diet or 2) Bt-MON810 maize diet, which were fed for ∼143 days throughout gestation and lactation. Immune function was assessed by leukocyte phenotyping, haematology and Cry1Ab-specific antibody presence in blood on days 0, 28 and 110 of gestation and at the end of lactation. Peripheral-blood mononuclear cell cytokine production was investigated on days 28 and 110 of gestation. Haematological analysis was performed on offspring at birth (n = 12/treatment). Presence of the *cry1Ab* transgene was assessed in sows' blood and faeces on day 110 of gestation and in blood and tissues of offspring at birth. Cry1Ab protein presence was assessed in sows' blood during gestation and lactation and in tissues of offspring at birth. Blood monocyte count and percentage were higher (*P*<0.05), while granulocyte percentage was lower (*P*<0.05) in Bt maize-fed sows on day 110 of gestation. Leukocyte count and granulocyte count and percentage were lower (*P*<0.05), while lymphocyte percentage was higher (*P*<0.05) in offspring of Bt maize-fed sows. Bt maize-fed sows had a lower percentage of monocytes on day 28 of lactation and of CD4^+^CD8^+^ lymphocytes on day 110 of gestation, day 28 of lactation and overall (*P*<0.05). Cytokine production was similar between treatments. Transgenic material or Cry1Ab-specific antibodies were not detected in sows or offspring.

**Conclusions/Significance:**

Treatment differences observed following feeding of Bt maize to sows did not indicate inflammation or allergy and are unlikely to be of major importance. These results provide additional data for Bt maize safety assessment.

## Introduction

The continuous increase in the area cultivated with genetically modified (GM) crops [1] and consequently their ubiquitous presence makes it increasingly difficult for consumers to avoid GM products. However, the dispute regarding the safety of GM crops is far from resolved [2] and consumer opinions vary over time and from country to country [3], [4].

Bt MON810 maize is resistant to attack by *Lepidopterae* pests, as it expresses the Cry1Ab protein [5]. This protein is produced in all plant tissues as a result of introduction of the bacterial *cry1Ab* transgene into the maize genome [6]. While the bacterial Cry1Ab protein has been extensively used as an organic insecticide [7], its expression in transgenic maize could potentially alter its structure which may render it allergenic or otherwise harmful upon ingestion [8]. Fears are expressed by consumers regarding the safety of transgenic compounds following long-term consumption [9], [10]. As pregnancy-related hormonal changes may result in immunosuppression [11], [12], the immune system of pregnant females may respond differently to dietary antigens. Maize is a major component of animal feed and the safety of feeding GM maize to breeding livestock is also of paramount importance.

Having been marketed in the US since 1996 and grown in market penetration since then [1] with no evidence to suggest harmful effects, Bt MON810 maize has a relatively long history of safe use [13]. Furthermore, numerous controlled studies have investigated the effects of dietary Bt maize in different animal species [14]. However, while several studies have investigated effects over multiple generations in rodents and ruminants [15], multi-generational studies in pigs are notably lacking from the literature. It is well known that the digestive physiology of pigs is very similar to that of humans [16]–[18]. Therefore, studies in pigs may provide some insight into the expected effects of trans-generational Bt maize consumption in humans, although the limitations of any animal model must always be taken into account.

The aim of the present study was to investigate the effects of feeding Bt MON810 maize to nulliparous sows during pregnancy and lactation on maternal and offspring immune function and to assess the presence of transgenic material in the blood of sows as well as in the blood and tissues of offspring at birth.

## Methods

### Ethical approval

The pig study complied with European Union Council Directives 91/630/EEC (outlines minimum standards for the protection of pigs) and 98/58/EC (concerns the protection of animals kept for farming purposes) and was approved by, and a license obtained from, the Irish Department of Health and Children (license number B100/4147). Ethical approval was obtained from Teagasc and Waterford Institute of Technology ethics committees.

### Maize and diets

Seeds derived from GM Bt MON810 and non-GM parent line control maize (PR34N44 and PR34N43, respectively; Pioneer Hi-Bred, Sevilla, Spain) were grown simultaneously side by side in 2007 in Valtierra, Navarra, Spain by independent tillage farmers. The Bt and non-Bt control maize were purchased by the authors from the tillage farmers for use in this animal study.

Diets were manufactured as previously described by Walsh et al. [19]. All diets were formulated to meet or exceed the National Research Council requirements for pigs of given weights [20]. The Bt and non-Bt control maize, as well as the whole diets, were sampled in accordance with international guidelines [21] and tested for chemical, carbohydrate and amino acid composition as well as for presence of pesticide contaminants, the *cry1Ab* transgene, and mycotoxins, as previously described by Walsh et al. [19].

### Animals and experimental design

Twenty four sows (Large White × Landrace) were purchased from Hermitage AI (Kilkenny, Ireland) as weanling pigs (∼28 days old) and raised to ∼165 kg on diets free of GM ingredients. On the day of insemination, sows were blocked by body weight and insemination date and randomly assigned to one of two dietary treatments: 1) non-Bt control parent line maize diet (Pioneer PR34N43) or 2) Bt maize diet (Pioneer PR34N44 event MON810). Sows were fed experimental diets from insemination throughout gestation and lactation until weaning at ∼28 days post-farrowing (∼143 days in total). Diets used in this animal study are presented in Table 1.

**Table 1:** Composition of diets fed to sows during gestation and lactation (fresh weight basis, %).

	Gestation		Lactation	
Ingredient	Non-Bt control	Bt	Non-Bt control	Bt
Maize	86.55	86.55	74.42	74.42
Soybean meal (non-GM)	10.33	10.33	19.30	19.30
Soybean oil	-	-	3.02	3.02
L-Lysine HCl	0.16	0.16	0.25	0.25
DL-Methionine	-	-	0.10	0.10
L-Threonine	-	-	0.06	0.06
Vitamin and mineral premix^1^	0.10	0.10	0.10	0.10
Salt	0.40	0.40	0.40	0.40
Di-calcium phosphate	1.36	1.36	1.29	1.29
Limestone flour	1.10	1.10	1.06	1.06
**Analysed chemical composition**				
Dry matter	88.40	87.80	89.50	88.60
Crude protein	11.80	11.00	15.60	15.00
Fat	3.00	3.20	5.90	6.00
Crude fibre	1.70	1.60	1.60	1.90
Ash	4.10	4.30	4.60	4.30
Lysine	0.64	0.68	1.01	0.96
Ca^2^	7.60	7.60	7.50	7.50
P^2^	6.10	6.10	6.20	6.20
Digestible energy, MJ of DE/kg^2^	13.80	13.80	14.50	14.50

1 Premix provided per kg of complete diet: Cu, 15 mg; Fe, 70 mg; Mn, 62 mg; Zn, 80 mg, I, 0.6 mg; Se, 0.2 mg; retinyl acetate, 3.44 mg; cholecalciferol, 25 μg; DL-alpha-tocopheryl acetate, 100 mg; vitamin K, 2 mg; vitamin B_12,_ 15 μg; riboflavin, 5 mg; nicotinic acid, 12 mg; pantothenic acid, 10 mg; choline chloride, 500 mg; vitamin B_1,_ 2 mg; and vitamin B_6,_ 3 mg.

2 Calculated values.

Synchronization of oestrus was achieved by administering 20 mg of altrenogest (Regumate™, Intervet/Schering-Plough Animal Health, Bray, Ireland) per gilt in the feed for 18 days. A sexually mature boar was housed adjacent to the gilts to stimulate oestrus. Nine days prior to predicted oestrus, the gilts were flush fed (4 kg/day) gilt developer diet (6.0 g/Kg lysine and 13.67 MJ/Kg DE). Sows were inseminated with pooled semen from five Hylean MaxGroTM boars (Hermitage AI) as soon as oestrus was detected and again 24 h later. Following insemination, sows were penned in individual gestation stalls (2.4 m ×0.6 m; O'Donovan Engineering, Coachford, Ireland) until day 110 of gestation. Environmental temperature was maintained between 20 and 22°C and sows were provided with *ad libitum* access to water from one drinker per pen (Arato, Köln, Germany). Supplementary feed was not offered to suckling piglets.

A vaccine to prevent *E. coli* neonatal enterotoxicosis in piglets (Porcoli Diluvac Forte™; Intervet/Schering-Plough Animal Health) was administered to all sows on days 74 and 99 of gestation. Zerofen™ (4% powder, Chanelle Animal Health Limited, Liverpool, UK) was administered to sows in feed (0.125 g/kg body weight) on day 110 of gestation for routine deworming.

From day 110 of gestation until weaning, sows were housed in three farrowing rooms with 10 farrowing crates (O'Donovan Engineering) per room. Control and treatment sows were allotted to farrowing rooms in equal numbers to minimise environmental influences. However, to minimise potential cross-contamination, control and treatment sows were penned together on either side of a central dividing passageway. Room temperature was maintained at 20°C and increased to 24°C for 48 h around farrowing. Sows had access to feed from a Daltec feeder (Daltec A/S, Egtved, Denmark) and were fed 2 kg/day immediately post-farrowing. Feed allowance was then increased by 500 g/day until day 7 post-farrowing when sows were offered *ad libitum* access to feed. Unlimited access to water was provided throughout lactation via a single nipple drinker per pen. Induction of farrowing was achieved by administering 2 mL of Enzaprost™ (5 mg/ml; CEVA Animal Health Ltd, Chesham Bucks, UK) on day 114 of gestation.

At farrowing, the fourth piglet born alive from each sow was euthanized before suckling occurred and samples were obtained, as outlined below.

At all times throughout the study sows were observed closely at least twice daily and any showing signs of ill health were treated as appropriate and all veterinary treatments recorded. Data from these animals was examined on a case by case basis and if necessary, removed from the data set.

### Blood, faeces and tissue sampling

Blood samples were taken from the external jugular vein of sows at insemination (day 0), at days 28 and 110 of gestation and on day 28 of lactation (n = 12/treatment). Blood samples were also collected from offspring at birth. Whole blood from sows and offspring was sampled in K_2_EDTA evacuated tubes (Vacuette, Greiner Bio One Ltd, Gloucestershire, UK) and stored at room temperature for whole blood haematological analysis (within 6 hours of sampling). Haematological analysis was performed using a Beckman Coulter Ac T Diff analyser (Beckman Coulter Ltd., High Wycombe, UK) for determination of white blood cell counts (WBC#) and counts and percentage of lymphocytes (LY# and LY%), monocytes (MONO# and MONO%) and granulocytes (GRAN# and GRAN%). Calibration of the analyser was performed in accordance with the manufacturer's instructions and accuracy was determined by testing control samples of known values.

Blood was also collected from sows in evacuated tubes containing a silica coagulation activator (Vacuette) and allowed to coagulate for 2 hours at room temperature. Serum was then collected following centrifugation at room temperature, at 1500×g for 10 minutes. The serum was analysed for presence of the Cry1Ab protein and its specific antibodies, as outlined below.

White blood cells for gene detection were isolated from sow and offspring blood collected in K_2_EDTA evacuated collection tubes. To prevent DNA degradation, the tubes were stored on ice following sampling and were centrifuged within 2 hours at 4°C, at 1500×g for 10 minutes. The separated plasma from offspring blood was also collected for Cry1Ab protein detection. White blood cell and plasma samples were stored at −20°C until analysed.

Blood samples were also collected from sows in sodium heparin tubes (BD Vacutainer Systems, Franklin Lakes, NJ, USA) for isolation of peripheral blood mononuclear cells (PBMC), as outlined below.

Prior to all blood sampling the skin at the sampling site was thoroughly swabbed with 70% ethanol to prevent contamination of blood samples with feed dust.

Colostrum samples for detection of Cry1Ab-specific antibodies (as outlined below) were collected from sows immediately prior to parturition in 30 ml sterile containers (Sterilin Limited, Newport, UK) and stored at −20°C until analysis. Sow teats were sanitised with 70% ethanol prior to sampling to prevent environmental contamination.

On day 110 of gestation, faecal samples were collected from sows and stored at −20°C prior to gene detection (n = 12/treatment).

The heart, kidney, spleen, liver, *semitendinosus* muscle, brain and navel tissue were collected from offspring at birth for Cry1Ab protein and transgene detection and stored at −20°C until analysed. Every effort was made to prevent environmental and cross-contamination; Scalpel blades and gloves were changed and all instruments were sanitised using 70% ethanol after each pig was sampled. Furthermore, each organ was placed on a single-use aluminium foil sheet to avoid cross-contamination and the outer surface was removed and samples were taken from an inner portion. In addition, to avoid contamination with feed dust, sampling of all tissues was performed in a room separate to the farrowing house.

### Immune cell populations and cytokine production

Sow PBMC were isolated and stimulated using a combination of phorbol 12-myristate 13-acetate and ionomycin on days 28 and 110 of gestation, as described by Walsh et al. [22]. Prior to and following stimulation, the cell culture supernatant was removed and stored at −80°C for analysis of cytokines (IL-4, IL-6, IL-8 and TNF-α). Cytokine concentrations were determined using multiplex porcine-specific ELISA kits (Meso Scale Discovery, Gaithersburg, MD, USA) in accordance with the manufacturer's instructions.

Staining of PBMC with fluorescent antibodies for flow cytometric analysis was also performed as described by Walsh et al. [22], [23] for determination of the percentages of white blood cells, B lymphocytes, monocytes and CD3^+^, CD4^+^, CD8^+^ and CD4^+^CD8^+^ T lymphocytes. Proportions of monocytes and B and T lymphocytes were calculated from the total number of white blood cells. Proportions of CD4^+^, CD8^+^ and CD4^+^CD8^+^ T lymphocytes were calculated from the number of CD3^+^ T lymphocytes. These cells were analysed in sows at day 0, 28 and 110 of gestation and at day 28 of lactation using a BD FACSCanto flow cytometer (BD Biosciences, Franklin Lake, NJ, USA) and FACSDiva software (BD Biosciences).

### Cry1Ab protein and transgene detection in faeces, blood and tissues

The presence of the Cry1Ab protein in the serum of sows at day 28 and 110 of gestation and day 28 of lactation and in the plasma and tissues of offspring euthanized at birth was assessed using the QuantiPlate kit for Cry1Ab/Cry1Ac detection (Envirologix, Maines, USA) in accordance with the manufacturer's instructions.

Cry1Ab protein was extracted from serum of sows and from organs (heart, kidney, spleen, liver, muscle, liver, navel cord and brain) of pigs euthanized at birth as previously described by Walsh et al. [24].

Extraction of DNA from sow and offspring white blood cells and offspring tissues (kidney, liver and muscle) was also conducted as previously outlined by Walsh et al. [23]. The presence of two *cry1Ab* transgene fragments (149 and 211 bp), the *sw* (porcine growth hormone) gene (108 bp), the *shrunken 2* gene *(sh2;* a maize-specific ADP glucose pyrophosphorylase gene; 213 bp) and the *rubisco* gene (maize-specific ribulose bisphosphate carboxylase; 173 bp) was assessed by PCR as previously described by Walsh et al. [23], [24].

### Cry1Ab-specific antibody response

The presence of Cry1Ab protein-specific immunoglobulins (IgA and IgG) was investigated in the serum at day 28 and 110 of gestation and day 28 of lactation and colostrum of sows and in the plasma of the offspring sacrificed at birth, as described by Walsh et al. [23].

### Statistical analysis

Statistical analysis was performed using SAS 9.2 [25]. To ensure normality, haematology data (except for sow GRAN% and offspring WBC# and LY# which were initially normally distributed) were transformed using the Box Cox transformation [26] x =  (y^λ^-1)/λ where y is the initial variable and λ is a constant (values of λ are presented in Tables 2 and 3). Data which were initially normally distributed or which were normalised using the Box Cox transformation were analysed using PROC MIXED, with treatment and day as fixed effects and block as a random effect. Day 0 values were used as a covariate in the model and day was included in the model as a repeated variable. Due to unequal spacing between sampling days and as indicated by the model fit criteria a spatial power covariance structure was fitted to the data [27]. The *slice* option was used to test for simple effects at each time point. The denominator degrees of freedom were computed using the Satterthwaite approximation. For analysis of offspring haematology, body weight was used as a covariate in the statistical model. To assess model suitability, data were examined using the influence diagnostics provided within PROC MIXED in SAS and by investigation of normality of scaled residuals using the Shapiro-Wilk test within the UNIVARIATE procedure in SAS. Least squares means were computed and *P* values were adjusted for multiple comparisons using the Tukey-Kramer adjustment. Means and 95% confidence limits for data which were normalised using the Box Cox transformation were back-transformed and are presented on the original scale. Data which were non-normal (cytokine production) were analysed using the non-parametric Kolmogorov-Smirnov test within PROC NPAR1WAY [28]. The *exact* option was used for non-parametric tests as recommended by Mundry and Fischer [29]. Non-parametric data is presented as medians and the 5^th^ and 95^th^ percentiles which were computed using PROC UNIVARIATE. Significance is reported for *P*≤0.05 and tendencies towards significance are reported for 0.05<*P*≤0.10. For all response criteria, the individual pig was the experimental unit.

**Table 2:** Effects of feeding Bt MON 810 maize to nulliparous sows during gestation and lactation on haematological parameters of sows^1^.

		Treatments				
	Transformation^2^	Non-Bt control		Bt		*P* – value
		Mean	95% CI^3^	Mean	95% CI^3^	
**WBC** 4 **, ×10** 3 **/µL**	−0.5					
Day 28 of gestation		20.4	18.3–23.0	21.3	19.0–24.0	0.61
Day 110 of gestation		16.2	14.7–18.0	16.4	14.9–18.3	0.90
Day 28 of lactation		18.0	16.2–20.1	17.8	16.0–19.8	0.83
Overall		18.0	16.9–19.3	18.3	17.1–19.6	0.82
**Lymphocytes, %**	1.75					
Day 28 of gestation		54.3	50.7–57.7	53.5	49.9–57.0	0.76
Day 110 of gestation		46.0	41.9–49.9	49.5	45.7–53.2	0.21
Day 28 of lactation		38.8	34.1–43.1	39.6	34.9–43.9	0.81
Overall		46.7	43.9–49.4	47.8	45.1–50.4	0.55
**Monocytes, %**	−0.5					
Day 28 of gestation		5.7	4.4–7.8	5.3	4.1–7.1	0.68
Day 110 of gestation		3.9	3.1–5.0	5.7	4.4–7.8	0.04
Day 28 of lactation		5.2	4.0–6.9	5.8	4.4–7.9	0.57
Overall		4.8	4.2–5.7	5.6	4.8–6.6	0.19
**Granulocytes, %**	none					
Day 28 of gestation		40.8	35.6–46.0	40.3	35.1–45.5	0.89
Day 110 of gestation		50.7	45.4–55.8	41.0	35.3–45.8	0.01
Day 28 of lactation		57.7	52.2–63.1	54.3	48.9–59.7	0.39
Overall		49.7	46.3–53.0	45.1	41.7–48.4	0.06
**Lymphocytes, ×10** 3 **/µL**	0.25					
Day 28 of gestation		10.9	10.0–11.9	11.6	10.6–12.7	0.34
Day 110 of gestation		7.3	6.6–8.0	8.2	7.4–9.0	0.09
Day 28 of lactation		7.0	6.3–7.8	7.0	6.3–7.7	0.93
Overall		8.3	7.8–8.8	8.8	8.3–9.3	0.14
**Monocytes, ×10** 3 **/µL**	−0.25					
Day 28 of gestation		1.2	0.9–1.6	1.2	0.9–1.6	0.89
Day 110 of gestation		0.6	0.5–0.8	1.0	0.8–1.4	0.03
Day 28 of lactation		0.9	0.7–1.2	1.0	0.8–1.4	0.38
Overall		0.9	0.7–1.0	1.1	0.9–1.3	0.09
**Granulocytes, ×10** 3 **/µL**	0.5					
Day 28 of gestation		8.6	7.0–10.4	8.8	7.2–10.6	0.88
Day 110 of gestation		8.3	6.7–10.0	6.5	5.1–8.0	0.11
Day 28 of lactation		10.4	8.6–12.3	9.7	8.0–11.5	0.57
Overall		9.1	7.9–10.3	8.3	7.2–9.5	0.33

1 Sows were fed either a non-Bt control or a Bt maize-based diet (n = 12/treatment).

2 Lambda used for Box Cox transformation of data to ensure normal distribution. Means and 95% confidence limits for data which were normalised using the Box Cox transformation were back transformed and are presented on the original scale.

3 95% CI –95% confidence interval (the interval that contains the true population mean in 95% of cases).

4 WBC – white blood cells.

**Table 3:** Effects of feeding Bt MON810 maize to nulliparous sows during gestation and lactation on offspring haematological parameters at birth^1^.

	Transformation^2^	Treatments				
		Non-Bt control		Bt		
		Mean	95% CI^3^	Mean	95% CI^3^	*P* – value
WBC^4^, ×10^3^/µL	none	5.0	3.9–6.1	3.4	2.4–4.5	0.05
Lymphocytes, %	1.5	60.6	47.9–72.2	76.6	65.4–87.0	0.05
Monocytes, %	0.75	15.7	11.7–19.9	13.4	9.6–17.5	0.33
Granulocytes, %	−0.25	11.6	6.0–25.3	4.7	2.8–8.8	0.01
Lymphocytes, ×10^3^/µL	none	2.9	2.2–3.7	2.4	1.7–3.2	0.34
Monocytes, ×10^3^/µL	−0.25	0.7	0.5–0.9	0.4	0.3–0.6	0.07
Granulocytes, ×10^3^/µL	−0.5	0.4	0.2–1.0	0.2	0.1–0.3	0.01

1 Sows were fed either a non-Bt control or a Bt maize-based diet and blood samples were collected at birth from the 4^th^ live born piglet from each litter (n = 12/treatment).

2 Value of lambda used for Box Cox transformation of data to ensure normal distribution. Means and 95% confidence limits for data which were normalised using the Box Cox transformation were back transformed and are presented on the original scale.

3 95% CI –95% confidence interval (the interval that contains the true population mean in 95% of cases).

4 WBC – white blood cells.

## Results

### Health of animals

One sow from the control treatment was observed to have a lack of appetite and fever at day 105 of gestation and received injectable penicillin between days 105 and 107 of gestation. Following treatment, the sow made a complete recovery. As all haematological and immunological parameters investigated for this sow had similar values to other sows from the same treatment, data from this sow were not removed from the data set.

### Maize and diets

The Bt maize and its non-Bt control counterpart had similar proximate and amino acid composition [19]. Likewise, amino acid content and proximate composition were similar for the non-Bt control and Bt diets used in the present study (Table 1).

### Effects of feeding Bt and non-Bt control maize to sows on sow and offspring haematology

Results of the haematological analysis are presented in Tables 2 and 3 for sows and offspring at birth, respectively. For sow haematology, no treatment × time interaction was observed (*P*>0.05; data not shown). WBC# was not different between treatments at any time point or overall (*P*>0.05). A time effect was observed for WBC#, with values decreasing up to day 100 of gestation and increasing thereafter (*P*<0.05; data not shown). Likewise, no treatment differences were observed for LY% overall or at individual time points. LY% decreased throughout the study (*P*<0.05; data not shown). On day 110 of gestation MONO% was higher in sows on the Bt treatment compared to sows on the control treatment (*P*<0.05). However, MONO% was not different between treatments at any other time point or overall (*P>*0.05*)* and no time effect was observed (*P*>0.05; data not shown). GRAN% was lower on day 110 of gestation (*P*<0.05) and, as a result, a tendency towards lower overall GRAN% (*P* = 0.06) was also observed for sows fed Bt maize diets compared to sows fed non-Bt control maize diets. GRAN% increased throughout the study (*P*<0.05; data not shown). LY# was not significantly different between treatments overall or at any time point (*P*>0.05). However, a tendency towards higher LY# on day 110 of gestation was observed in sows fed Bt maize diets compared to sows fed non-Bt control maize diets (*P* = 0.09). LY# decreased throughout the study (*P*<0.05; data not shown). MONO# was higher on day 110 of gestation (*P*<0.05) and this lead to a tendency towards a higher overall MONO# in sows on the Bt treatment compared to the control treatment (*P* = 0.09). A time effect was also observed for MONO#, with values decreasing up to day 110 of gestation and increasing thereafter (*P*<0.05; data not shown). No treatment effects were observed for GRAN# (*P*>0.05); however, values decreased up to day 110 of gestation and thereafter increased up to day 28 of lactation (*P*<0.05; data not shown).

At birth, WBC# was lower (*P*≤0.05) and LY% was higher (*P*≤0.05) for offspring of Bt maize-fed sows. GRAN% and GRAN# were lower for offspring of sows fed Bt maize (*P*<0.05). MONO# tended to be lower for offspring of sows fed Bt maize (*P* = 0.07). No treatment effects were observed for MONO% and LY# in offspring at birth (*P*>0.05).

### Effects of feeding Bt and non-Bt control maize to sows on immune cell populations from isolated peripheral blood mononuclear cells

The results from flow cytometric analysis of immune cell populations present in peripheral blood are presented in Table 4. No treatment effects were observed for the percentage of white blood cells. However, a treatment × time interaction (*P*<0.05; data not shown) and a time effect (*P*<0.05; data not shown) were observed, with white blood cells increasing up to day 110 of gestation and decreasing thereafter. Similar values were observed for both treatments at all time points and overall for the percentage of peripheral blood B lymphocytes (*P*>0.05). B lymphocyte percentage increased up to day 110 of gestation and decreased thereafter (*P*<0.05; data not shown). A treatment × time interaction (*P*<0.05; data not shown) was observed for monocyte percentage and values increased over time (*P*<0.05). On day 28 of lactation the Bt maize-fed sows had lower peripheral blood monocyte percentages than non-Bt control maize-fed sows (*P*<0.05). No treatment effects were observed for the percentages of CD3^+^ T lymphocytes and CD4^+^ T lymphocytes (*P*>0.05); however, both increased up to day 110 of gestation and then decreased up to day 28 of lactation (*P*<0.05; data not shown). CD8^+^ T lymphocyte percentages in peripheral blood were not affected by feeding Bt maize (*P*>0.05); however, values decreased throughout the study (*P*<0.05; data not shown). A decrease in CD4^+^CD8^+^ T lymphocyte counts was observed for sows on the Bt treatment compared to sows on the control treatment on day 110 of gestation, day 28 of lactation and overall (*P*<0.05). Likewise, the percentage of CD4^+^CD8^+^ T lymphocytes in peripheral blood increased over time (*P*<0.05; data not shown).

**Table 4:** Effects of feeding Bt MON 810 maize to nulliparous sows during gestation and lactation on peripheral blood immune cell populations^1,2^.

	Treatments			
	Non-Bt control	Bt	SEM^3^	*P* – value
**WBC** 4				
d 28 of gestation	42.1	40.5	3.88	0.84
d 110 of gestation	57.1	50.4	3.04	0.28
d 28 of lactation	46.0	52.3	3.07	0.31
Overall	48.4	47.7	3.79	0.90
**B lymphocytes**				
d 28 of gestation	2.8	2.0	0.47	0.42
d 110 of gestation	4.4	4.0	0.35	0.53
d 28 of lactation	3.4	2.9	0.36	0.54
Overall	3.5	3.0	0.36	0.29
**Monocytes**				
d 28 of gestation	9.1	6.8	1.25	0.37
d 110 of gestation	13.5	15.8	0.95	0.25
d 28 of lactation	19.1	13.5	0.95	0.01
Overall	13.9	12.1	0.94	0.17
**CD3^+^ T lymphocytes**				
d 28 of gestation	51.8	52.0	2.44	0.97
d 110 of gestation	60.6	63.1	1.86	0.52
d 28 of lactation	58.1	60.5	1.83	0.53
Overall	56.8	58.5	1.97	0.57
**CD4^+^ T lymphocytes**				
d 28 of gestation	7.3	7.4	1.89	0.99
d 110 of gestation	21.9	22.5	1.33	0.82
d 28 of lactation	22.3	19.3	1.35	0.28
Overall	17.2	16.4	1.44	0.70
**CD8^+^ T lymphocytes**				
d 28 of gestation	38.4	40.0	1.91	0.68
d 110 of gestation	36.1	40.7	1.36	0.11
d 28 of lactation	22.2	24.9	1.38	0.33
Overall	32.2	35.2	1.59	0.20
**CD4^+^CD8^+^ T lymphocytes**				
d 28 of gestation	12.9	11.5	1.45	0.56
d 110 of gestation	19.5	15.0	1.15	0.01
d 28 of lactation	19.3	15.4	1.15	0.03
Overall	17.2	14.0	1.17	0.02

1 Sows were fed either a non-Bt control or a Bt maize-based diet (n  = 12/treatment).

2 Values are given as percentages of the total peripheral blood mononuclear cell population for all parameters, except the CD4^+^ CD8^+^ and CD4^+^CD8^+^ lymphocytes which are given as percentages of the CD3^+^ lymphocytes.

3 SEM – standard error of the mean.

4 WBC – white blood cells.

### Effects of feeding Bt and non-Bt control maize on cytokine production from isolated PBMC

Cytokine production data from resting and stimulated PBMC are presented in Table 5. No treatment effect was observed for IL-4 and IL-6 production by resting PBMC on day 28 or 110 of gestation (*P*>0.05). However, production of both IL-4 and IL-6 by resting cells increased over time (*P*<0.05; data not shown). A tendency towards lower production of IL-8 by resting PBMC was observed on day 28 and 110 of gestation in sows fed Bt maize (*P* = 0.10). No time effect was observed for IL-8 (*P*>0.05; data not shown). TNF-α production by resting PBMC was not affected on day 28 of gestation (*P*>0.05); however, a tendency towards lower TNF-α production was observed in Bt maize-fed sows on day 110 of gestation (*P* = 0.10). The production of TNF-α by resting PBMC increased over time (*P*<0.05; data not shown).

**Table 5:** Effects of feeding Bt MON810 maize to nulliparous sows during gestation on cytokine production from resting and stimulated peripheral blood mononuclear cells (pg/mL)^1^.

	Non-Bt control	5–95^th^ percentiles^2^	Bt	5–95^th^ percentiles^2^	*P* - value^3^
***Resting***					
**Day 28 of gestation**					
IL-4	0.5	0–8.1	0.1	0–5.2	0.77
IL-6	1.2	0–14.1	0.7	0–3.2	0.52
IL-8	1666.8	66.6–10795.5	1150.3	278.7–1506.4	0.10
TNF-α	10.5	0.4–71.7	8.5	3.0–36.2	0.54
**Day 110 of gestation**					
IL-4	108.0	0–1049.4	72.8	0–121.9	0.24
IL-6	19.9	0–1184.6	22.8	0–45.5	0.85
IL-8	3691.7	0–21749.2	1233.0	0–4127.1	0.10
TNF-α	222.1	0–25555.2	105.1	16.2–319.2	0.10
***Stimulated***					
**Day 28 of gestation**					
IL-4	12.8	0–127.2	16.9	1.3–45.1	0.87
IL-6	38.5	9.3–203.3	34.3	14.3–198.7	0.99
IL-8	6527.7	1742.0–13819.8	6801.2	1703.5–18474.8	0.87
TNF-α	9331.6	3056.7–28898.4	11725.9	3519.3–23946.0	0.87
**Day 110 of gestation**					
IL-4	447.0	0–1227.4	457.9	300.4–656.5	0.87
IL-6	400.3	0–753.7	475.9	260.1–672.5	0.87
IL-8	11757.8	0–36328.5	11882.7	8010.8–15270.9	0.54
TNF-α	18765.3	133.2–130654.7	18185.9	12167.8–22784.5	0.54

1 Sows were fed either a non-Bt control or a Bt maize-based diet (n  = 12/treatment).

2 The 5^th^ percentile is larger than 5% of the values and the 95^th^ percentile is larger than 95% of the values.

3 Computed on untransformed data using the Kolmogorov-Smirnov non-parametric test within PROC NPAR1WAY in SAS.

The production of IL-4, IL-6, IL-8 or TNF-α by stimulated PBMC was not different between treatments at any time point (*P*>0.05). However, values for all cytokines increased over time (*P*<0.05; data not shown). The production of all cytokines increased with mitogen stimulation (*P*<0.05; data not shown).

### Cry1Ab protein and transgene detection in blood, faeces and tissues

The Cry1Ab protein was not detected in the serum of sows at day 28 and 110 of gestation or at the end of lactation (data not shown) or in the plasma, heart, kidney, spleen, muscle or brain of offspring from sows fed either non-Bt control or Bt maize throughout gestation (data not shown).

The frequency of detection of both transgenic and maize and porcine endogenous gene fragments is presented in Tables 6 and 7 for sows and offspring at birth, respectively. The 108 bp gene fragment of the pig-specific *sw* gene was detected in white blood cells of all sows from both treatments on day 110 of gestation. The 173 bp fragment of the maize-specific multi-copy *rubisco* gene was detected in the white blood cell samples of 2 of 12 sows fed the non-Bt control maize diet and in 1 of 12 sows fed the Bt maize diet. In faeces, the same gene fragment was detected in 7 and 5 of 12 sows on the non-Bt control and Bt treatments, respectively. The 213 bp fragment of the maize-specific single-copy *sh2* gene was not detected in white blood cells or faeces of sows from either treatment. The single-copy 149 or 211 bp fragments of the *cry1Ab* transgene were not detected in the blood or faeces of sows from either treatment group.

**Table 6:** Detection of endogenous maize and porcine gene fragments and transgene fragments in blood and faeces of sows fed non-Bt control or Bt maize-based diets during gestation and lactation^1^.

	Treatments			
	Non-Bt control	Bt	Non-Bt control	Bt
Gene (fragment length)	White blood cells		Faeces	
**Endogenous**				
*sw* – pig-specific (108 bp)	12	12	NA^2^	NA^2^
*rubisco* – multicopy maize-specific (173 bp)	2	1	7	5
*sh2* – single copy maize-specific (213 bp)	0	0	0	0
**Transgenic (single copy)**				
*cry1Ab* (149 bp)	0	0	0	0
*cry1Ab* (211 bp)	0	0	0	0

1 Number of samples out of 12 that tested positive for the gene fragment of interest. One sample was tested per sow (n  = 12 sows/treatment).

2 Not assessed.

**Table 7:** Detection of endogenous maize and porcine gene fragments and transgene fragments in blood and organs of offspring from sows fed non-Bt control or Bt maize-based diets during gestation and lactation^1,2^.

	Treatments								
	Non-Bt control	Bt	Non-Bt control	Bt	Non-Bt control	Bt	Non-Bt control	Bt	
Gene (fragment length)	White blood cells		Kidney		Liver		Muscle		
**Endogenous**									
*sw* – pig specific (108 bp)	12	11	12	12	12	12	12	12	
*rubisco* – multicopy maize specific (173 bp)	2	2	0	0	0	0	1	1	
*sh2* – single copy maize specific (213 bp)	0	0	0	0	0	0	0	0	
**Transgenic (single copy)**									
*cry1Ab* (149 bp)	0	0	0	0	0	0	0	0	
*cry1Ab* (211 bp)	0	0	0	0	0	0	0	0	

1 Samples were collected at birth from the 4^th^ live born piglet from each litter.

2 Number of samples out of 12 that tested positive for the gene fragment of interest. One sample was tested per piglet (n = 12 piglets/treatment).

In the offspring at birth, the pig-specific *sw* gene fragment was detected in all white blood cell samples from the control treatment and in 11 of 12 white blood cells samples from the Bt treatment. The *sw* gene fragment was also detected in all kidney, liver and muscle samples from both treatment groups. The maize-specific multi-copy *rubisco* gene fragment was present in the white blood cells of two animals from each treatment group and in the muscle of one animal from each treatment group but was not detected in any kidney or liver samples analysed. The maize-specific single-copy *sh2* gene fragment was not detected in any of the white blood cell, kidney, liver or muscle samples from either the control or Bt treatment group. Neither the 149 bp nor the 211 bp transgene fragments were detected in white blood cells, kidney, liver or muscle of offspring at birth from either non-Bt control or Bt maize-fed sows.

### Cry1Ab-specific antibody response

Neither Cry1Ab-specific IgA nor IgG were detected in serum from sows at any sampling point, in colostrum taken from sows immediately prior to parturition or in plasma from offspring from either treatment group (data not shown).

## Discussion

Most studies that have previously investigated the safety of GM feed ingredients with respect to animal health have generally involved only one generation of animals. For this reason multi-generational studies are needed to fully establish the safety of feeding GM feed ingredients over multiple generations. To our knowledge, this is the first study to investigate immune function and to assess the presence of transgenic products in blood and tissues of Bt maize-fed pregnant sows and their offspring. Although there are no data to suggest that the Cry1Ab protein is toxic to mammals (most likely because they lack specific receptors in the intestinal tract [5]), nonetheless consumers are concerned about potential toxicity [2]. However, Cry1Ab occurs in relatively low quantities in Bt maize (e.g. 0.6 μg/g in this study). Furthermore, incorporation of Bt maize in complete animal feedstuffs results in dilution. For these reasons, subtle effects may not be evident unless multiple health indicators are examined following high-level long-term exposure [15], [30]. Therefore, in the present study, Bt maize was included at high levels in pig diets (74–87%), and the duration of the study was extended beyond the 90-day standard recommended by the European Food Safety Authority [31]. In addition, a wide array of health indicators was assessed in sows and their offspring to determine the long-term and trans-generational safety of feeding Bt maize.

Food allergies are characterised by intestinal inflammation and are associated with diarrhoea and malabsorption [32], [33]. However, no such symptoms were observed in pregnant sows fed Bt maize [34]. Prolonged allergen exposure increases the magnitude of an inflammatory response and compromises the integrity of the intestinal epithelium, thereby allowing the allergen to enter the circulatory system which may lead to development of a systemic immune response [32], [35], [36]. Previous studies have demonstrated that the quantity of Cry1Ab protein that survives intestinal digestion is very low and have also confirmed that the Cry1Ab protein is absent from the blood and organs of livestock fed Bt maize for extended periods [23], [24], [37]–[40]. However, even at low doses, the Cry1Ab protein has the potential to be allergenic, as, in general, only minute quantities of allergens are required for an allergic response in sensitized individuals [36].

In the present study, an increase in the proportion of circulating T lymphocytes and, more specifically, an increase in CD4^+^ T or B lymphocytes, which would be indicative of allergy [36] were not observed at any time point. The decrease in monocytes and in immature CD4^+^CD8^+^ T lymphocytes from PBMC observed at day 28 of lactation in response to feeding Bt maize to sows is unlikely to denote an inflammatory/allergic response. This is because only an increase of these cell populations associated with an increase in B and CD4^+^ T lymphocytes and accompanied by production of Cry1Ab-specific antibodies would indicate such a response [33], [36]. An increase in the cytokines involved in allergic/inflammatory responses, such as IL-4, IL-6 IL-8 and TNF-α [41], [42], was also not observed at any time point during the study. Furthermore, Cry1Ab-specific IgG and IgA were not detected in response to Bt maize feeding, making it unlikely that treatment differences observed would indicate an allergic or inflammatory response. The absence of treatment differences for cytokine production by stimulated PBMC also indicates that Bt maize-fed animals are likely to respond in a similar fashion in an *in-vivo* challenge situation. These results are in agreement with our previous findings in pigs fed Bt maize for 31 [23] or 110 days [24], where no differences in IL-4, IL-6, IL-8 and TNF-α production were found between treatments in either resting or stimulated PBMC. In contrast, differences in cytokine production and immune cell populations were observed in mice fed Bt maize for 30 or 90 days compared to controls [43]. Although the biological significance of those findings was questioned by the authors, the responses observed were believed to be as result of a higher quantity of the maize allergen zein due to *cry1Ab* insertion [43]. While this may be true, environment, growing location and developmental stage have all been shown to have a greater influence on maize allergens and metabolites than the genetic modification itself [44]–[46].

An inflammatory response is also characterised by an increased white blood cell count and increased granulocytes and monocytes (which are both white blood cell types), representing the innate immune response. This is then followed by an increase in lymphocytes during the development of the adaptive immune response [36]. On the contrary, in the present study, the proportion of monocytes within the PBMC decreased on day 28 of lactation in response to feeding Bt maize, although this was not reflected in the findings for monocytes within whole blood. In fact, an increase in monocytes was observed during haematological analysis of Bt maize-fed sows during gestation. This could be explained by the higher body weight observed in these sows in the second half of gestation [34], as monocyte recruitment and increased cytokine production are known to be associated with an increase in body weight [47]. However, no differences in fat deposition or cytokine production were observed between treatments. Furthermore, monocyte counts remained within the normal range for pigs and this difference did not persist up to day 28 of lactation. While an increase in granulocyte percentage could be indicative of inflammation, in the present study we observed only a decrease in granulocyte percentage in whole blood for Bt maize-fed sows on day 110 of gestation, which was mirrored in the blood of offspring at birth. A lower abundance of the potentially pathogenic *Proteobacteria* observed in faeces of Bt maize-fed sows (Buzoianu et al., unpublished) may be the cause of the lower granulocytes and tendencies towards lower IL-8 and TNF-α observed in these sows, as *Proteobacteria* have been positively correlated with increased blood granulocytes and cytokine production [48], [49]. Illness was not the reason for the treatment differences in immune cell populations observed in sows, as all animals were in good health, except for one sow in which immune parameters were unaffected (as outlined above). Likewise, the variability between animals on the same treatment was small, as evidenced by the confidence intervals. While no differences were observed for total white blood cell counts in sows fed Bt maize, a lower WBC# was observed in their offspring at birth. Only an increase in WBC# is associated with an allergic or inflammatory response. However, a higher LY% was observed at birth in the blood of offspring of Bt maize-fed sows and lymphocytes are a subset of white blood cells, but this was not mirrored in sows. The absence of the Cry1Ab protein in sow or foetal blood indicates that there was no contact between the Cry1Ab and the foetal immune system which was also evidenced by the absence of Cry1Ab-specific antibodies in blood of either sows or their offspring. Furthermore, an increase in spleen weight which is known to accompany a systemic immune response [50] was not observed in these piglets at birth [34]. An additional study using littermates of piglets from the present study found no increase in spleen weight later in life (∼143 days of age) and an absence of health abnormalities (Buzoianu et al, unpublished). Therefore, while treatment differences were observed in offspring at birth for certain haematological parameters in this study, they are not believed to indicate an allergic/inflammatory response to Bt maize ingestion by the sows. Furthermore, the values in sows generally remained within the normal range reported for pigs [50]. However, in offspring, lymphocyte and monocyte percentages were higher and granulocyte count and percentage were lower than the normal ranges [50], [51]. Stress is known to have a major influence on haematological parameters [50] and as these samples were taken immediately after birth, a time of great stress for the piglet, this may help to explain the deviations from the normal ranges.

Differences in immune response have previously been observed in sheep following Bt maize consumption for three years [52] and in mice after 30 and 90 days of Bt maize consumption [43]. Similar to results from our study, biological significance of these findings was also questioned by the authors and the inconsistency in results between studies is likely to be due to the use of different animal models. However, while neither of the above studies investigated Cry1Ab-specific antibody production, our results, as well as those of Adel-Patient et al. [53] for mice, indicate that no antibodies to the Cry1Ab protein are produced as a result of oral exposure to Bt MON810 maize.

Although present in the diet, both transgenic and native single-copy gene fragments (*cry1Ab* and *sh2*) were degraded along the gastrointestinal tract (GIT), as they were not detected in the faeces of sows or in the organs of the offspring. However, the multicopy *rubisco* maize gene was present in sow faeces and was detected at low frequency in the blood and in the muscle and blood of offspring, but did not reach the organs. Extensive DNA degradation was expected, as high DNase activity is found along the porcine GIT [18], [54]. In agreement with our previous results [23], [24] and those of others [37], [40], [52], [55], the present study confirms that DNA from single-copy genes is degraded along the GIT of livestock and does not reach the organs. There is no clear explanation why Mazza et al. [56] detected plant genes (both endogenous and transgenic) in the organs of 35 kg pigs, but discrepancies may be related to age/weight of the pigs, maturity of the intestine, dietary inclusion rate and time since the last meal. While DNA transfer to tissues and across the placenta has been confirmed in the present study, the frequency of detection appears to be more related to the number of copies of the gene of interest than to its endogenous or transgenic nature. Our results confirm that there are no differences in degradability and uptake of transgenic DNA compared to that of native plant DNA. In addition, incorporation and stable expression of transgenic DNA into the host genome and detrimental effects on the host as a consequence have not been demonstrated.

The fact that responses to Bt maize consumption are inconsistent across different species highlights inter-species variability and calls into question the relevance of these findings to humans. Therefore, the biological relevance of findings in the animal model should first be addressed before attempting to extrapolate results to humans. Furthermore, as biological processes are complex and interrelated in nature, investigating a wide range of parameters simultaneously allows for a more definitive assessment of biological relevance. Ruminants and rodents are frequently used as models for humans; however, they are not without shortcomings [57], [58]. As porcine intestinal physiology and microbiota as well as aspects of immune function are similar to that of humans [16], [17], [57], pigs are increasingly used as models for human immune response and even as candidates for xenotransplantation [32], [59], [60]. Therefore, our results in pigs, which demonstrate a lack of biologically significant effects on immune function as a result of feeding Bt maize could potentially be considered of more relevance to humans than findings from studies which use less appropriate animal models.

Overall, this study provides additional safety data which demonstrates that Bt maize is unlikely to pose any risk to pig health, even following long-term consumption and a similar response could be expected in humans. While differences in a limited number of immune parameters were observed in breeding pigs and their offspring in response to maternal intake of Bt maize, we consider these differences insufficient to indicate consistent activation of the innate immune system. Likewise, activation of the adaptive immune system (Th2 profile/allergy or Th1 profile/inflammation) was not observed in the present study. Furthermore, cytokine production was neither significantly different between treatments nor indicative of an immune response to Bt maize consumption. As neither the Cry1Ab protein nor antibodies specific to it were detected in the blood of either sows or offspring, these results support the conclusion that feeding Bt maize to pregnant sows during gestation and lactation does not adversely affect maternal or foetal immune function.

## References

[1] James C (2010) Global status of commercialized biotech/GM crops: 2010. ISAAA Brief No. 42. Ithaca, NY, USA. 241 p.

[2] Dona A, Arvanitoyannis IS (2009) Health risks of genetically modified foods. Crit Rev Food Sci Nutr 49: 164–175. 18989835 10.1080/10408390701855993

[3] Gaskell G (2005) Europeans and biotechnology in 2005: patterns and trends. A report to the European Commission's Directorate-General for Research.

[4] Gaskell G, Stares S, Allansdottir A, Allum N, Castro P, et al. (2010) EUR24537 - Europeans and biotechnology in 2010 winds of change? Luxembourg: Publications Office of the European Union. 172 p.

[5] Schnepf E, Crickmore N, Van Rie J, Lereclus D, Baum J, et al (1998) *Bacillus thuringiensis* and its pesticidal crystal proteins. Microbiol Rev 62: 775–806. 10.1128/mmbr.62.3.775-806.1998 PMC98934 9729609

[6] Hill M, Launis K, Bowman C, McPherson K, Dawson J, et al (1995) Biolistic introduction of a synthetic *Bt* gene into elite maize. Euphytica 85: 119–123.

[7] Betz FS, Hammond BG, Fuchs RL (2000) Safety and advantages of *Bacillus thuringiensis*-protected plants to control insect pests. Regul Toxicol Pharmacol 32: 156–173. 11067772 10.1006/rtph.2000.1426

[8] Prescott VE, Hogan SP (2006) Genetically modified plants and food hypersensitivity diseases: Usage and implications of experimental models for risk assessment. Pharmacol Ther 111: 374–383. 16364445 10.1016/j.pharmthera.2005.10.005

[9] Wal JM, Hepburn PA, Lea LJ, Crevel RWR (2003) Post-market surveillance of GM foods: applicability and limitations of schemes used with pharmaceuticals and some non-GM novel foods. Regul Toxicol Pharmacol 38: 98–104. 12878059 10.1016/s0273-2300(03)00079-5

[10] EFSA (2008) Safety and nutritional assessment of GM plants and derived food and feed: The role of animal feeding trials. Food Chem Toxicol 46: S2–S70. 10.1016/j.fct.2008.02.008 18328408

[11] Poole J, Claman H (2004) Immunology of pregnancy. Clin Rev Allergy Immunol 26: 161–170. 15208462 10.1385/CRIAI:26:3:161

[12] Butts CL, Sternberg EM (2008) Neuroendocrine factors alter host defense by modulating immune function. Cell Immunol 252: 7–15. 18329009 10.1016/j.cellimm.2007.09.009 PMC2590632

[13] EFSA (2009) Scientific opinion of the panel on genetically modified organisms on applications (EFSA-GMO-RX-MON810) for the renewal of authorisation for the continued marketing of (1) existing food and food ingredients produced from genetically modified insect resistant maize MON810; (2) feed consisting of and/or containing maize MON810, including the use of seed for cultivation; and of (3) food and feed additives, and feed materials produced from maize MON810, all under Regulation (EC) No 1829/2003 from Monsanto. The EFSA Journal 1149: 1–85.

[14] Domingo JL, Giné Bordonaba J (2011) A literature review on the safety assessment of genetically modified plants. Environ Int 37: 734–742. 21296423 10.1016/j.envint.2011.01.003

[15] Snell C, Bernheim A, Berge J-B, Kuntz M, Pascal G, et al (2012) Assessment of the health impact of GM plant diets in long-term and multigenerational animal feeding trials: A literature review. Food Chem Toxicol 50: 1134–1148. 22155268 10.1016/j.fct.2011.11.048

[16] Moughan PJ, Birtles MJ, Cranwell PD, Smith WC, Pedraza M (1992) The piglet as a model animal for studying aspects of digestion and absorption in milk-fed human infants. World Rev Nutr Diet 67: 40–113. 1557912 10.1159/000419461

[17] Kararli TT (1995) Comparison of the gastrointestinal anatomy, physiology, and biochemistry of humans and commonly used laboratory animals. Biopharm Drug Dispos 16: 351–380. 8527686 10.1002/bdd.2510160502

[18] Lewis AJ, Southern LL, editors (2001) Swine Nutrition. 2^nd^ Edition ed. Boca Raton, FL, USA: CRC Press. 1009 p.

[19] Walsh MC, Buzoianu SG, Gardiner GE, Cassidy JP, Rea MC, et al (2012) Effects of short-term feeding of Bt MON810 maize on growth performance, organ morphology and function in pigs. Br J Nutr 107: 364–371. 21733303 10.1017/S0007114511003011

[20] NRC (1998) Nutrient requirements of swine. Washington, DC, USA: National Academy of Sciences. 189 p.

[21] Hartnell GF, Cromwell GL, Dana GR, Lewis AJ, Baker DH, et al. (2007) Best practices for the conduct of animal studies to evaluate crops genetically modified for output traits; International Life Sciences Institute, editor. Washington, DC, USA. 194 p.

[22] Walsh MC, Gardiner GE, Hart OM, Lawlor PG, Daly M, et al (2008) Predominance of a bacteriocin-producing *Lactobacillus salivarius* component of a five-strain probiotic in the porcine ileum and effects on host immune phenotype. FEMS Microbiol Ecol 64: 317–327. 18373687 10.1111/j.1574-6941.2008.00454.x

[23] Walsh MC, Buzoianu SG, Gardiner GE, Rea MC, Gelencsér E, et al. (2011) Fate of transgenic DNA from orally administered Bt MON810 maize and effects on immune response and growth in pigs. PLoS ONE 6: 11 e27177. 10.1371/journal.pone.0027177 PMC3223173 22132091

[24] Walsh MC, Buzoianu SG, Rea MC, O'Donovan O, Gelencsér E, et al (2012) Effects of feeding Bt MON810 maize to pigs for 110 days on peripheral immune response and digestive fate of the *cry1ab* gene and truncated Bt toxin. PLoS ONE 7: e36141. 22574138 10.1371/journal.pone.0036141 PMC3345032

[25] SAS/STAT 9.22 (2010) User's Guide. Cary, NC, USA: SAS Institute Inc.

[26] Osborne JW (2010) Improving your data transformations: applying the Box-Cox transformation. Practical Assessment, Research & Evaluation 15.

[27] Littell RC, Milliken GA, Stroup WW, Wolfinger RD (1996) SAS system for mixed models. Cary, NC, USA: SAS Institute. 633 p.

[28] Pappas PA, DePuy V (2004) An overview of non-parametric tests in SAS: when, why, and how. SESUG 2004: The Proceedings of the SouthEast SAS Users Group, Nashville,TN 2004: TU04.

[29] Mundry R, Fischer J (1998) Use of statistical programs for nonparametric tests of small samples often leads to incorrect *P* values: examples from Animal Behaviour. Anim Behav 56: 256–259. 9710485 10.1006/anbe.1998.0756

[30] de Vendômois J, Cellier D, Vélot C, Clair E, Mesnage R, et al (2010) Debate on GMOs health risks after statistical findings in regulatory tests. Int J Biol Sci. 6: 590–598. 10.7150/ijbs.6.590 PMC2952409 20941377

[31] EFSA (2011) Guidance for risk assessment of food and feed from genetically modified plants. The EFSA Journal 9: 2150.

[32] Helm RM, Furuta GT, Stanley JS, Ye J, Cockrell G, et al (2002) A neonatal swine model for peanut allergy. J Allergy Clin Immunol 109: 136–142. 11799380 10.1067/mai.2002.120551

[33] Bailey M, Haverson K (2006) The postnatal development of the mucosal immune system and mucosal tolerance in domestic animals. Vet Res 37: 443–453. 16611557 10.1051/vetres:2006013

[34] Walsh MC, Buzoianu SG, Gardiner GE, Rea MC, O'Donovan O, et al. (in press) Effects of feeding Bt MON810 maize to sows during first gestation and lactation on maternal and offspring health indicators. Br J Nutr DOI 10.1017/S0007114512002607. 10.1017/S0007114512002607 23168255

[35] Helm RM, Burks AW (2000) Mechanisms of food allergy. Curr Opin Immunol 12: 647–653. 11102767 10.1016/s0952-7915(00)00157-6

[36] Janeway CA, Travers P, Walport M, Shlomchick Mark J, editors (2001) Immunobiology: the immune system in health and disease. NY: Garland Publishing. 732 p.

[37] Chowdhury EH, Kuribara H, Hino A, Sultana P, Mikami O, et al (2003) Detection of corn intrinsic and recombinant DNA fragments and Cry1Ab protein in the gastrointestinal contents of pigs fed genetically modified corn Bt11. J Anim Sci 81: 2546–2551. 14552382 10.2527/2003.81102546x

[38] Einspanier R, Lutz B, Rief S, Berezina O, Zverlov V, et al (2004) Tracing residual recombinant feed molecules during digestion and rumen bacterial diversity in cattle fed transgene maize. Eur Food Res Technol 218: 269–273.

[39] Lutz B, Wiedemann S, Einspanier R, Mayer J, Albrecht C (2005) Degradation of Cry1Ab protein from genetically modified maize in the bovine gastrointestinal tract. J Agric Food Chem 53: 1453–1456. 15740023 10.1021/jf049222x

[40] Wiedemann S, Lutz B, Albrecht C, Kuehn R, Killermann B, et al (2009) Fate of genetically modified maize and conventional rapeseed, and endozoochory in wild boar (*Sus scrofa*). Mamm Biol 74: 191–197.

[41] Reddy NRJ, Borgs P, Wilkie BN (2000) Cytokine mRNA expression in leukocytes of efferent lymph from stimulated lymph nodes in pigs. Vet Immunol Immunopathol 74: 31–46. 10760388 10.1016/s0165-2427(00)00164-1

[42] Murtaugh MP, Foss DL (2002) Inflammatory cytokines and antigen presenting cell activation. Vet Immunol Immunopathol 87: 109–121. 12072225 10.1016/s0165-2427(02)00042-9

[43] Finamore A, Roselli M, Britti S, Monastra G, Ambra R, et al (2008) Intestinal and peripheral immune response to MON810 maize ingestion in weaning and old mice. J Agric Food Chem 56: 11533–11539. 19007233 10.1021/jf802059w

[44] Barros E, Lezar S, Anttonen MJ, van Dijk JP, Röhlig RM, et al (2010) Comparison of two GM maize varieties with a near-isogenic non-GM variety using transcriptomics, proteomics and metabolomics. Plant Biotechnol J 8: 436–451. 20132517 10.1111/j.1467-7652.2009.00487.x

[45] Batista R, Oliveira M (2010) Plant natural variability may affect safety assessment data. Regulatory Toxicology and Pharmacology 58: S8–S12. 20804807 10.1016/j.yrtph.2010.08.019

[46] Fonseca Ct, Planchon S, Renaut J, Oliveira MM, Batista R (2012) Characterization of maize allergens - MON810 vs. its non-transgenic counterpart. J Proteomics 75: 2027–2037. 22270010 10.1016/j.jprot.2012.01.005

[47] Shoelson SE, Lee J, Goldfine AB (2006) Inflammation and insulin resistance. J Clin Invest 116: 1793–1801. 16823477 10.1172/JCI29069 PMC1483173

[48] Szczepanik M (2006) Interplay between *Helicobacter pylori* and the immune system. Clinical implications. J Physiol Pharmacol 57 Suppl 3: 15–27. 17033103

[49] Mukhopadhya I, Hansen R, El-Omar EM, Hold GL (2012) IBD-what role do *Proteobacteria* play? Nat Rev Gastroenterol Hepatol 9: 219–230. 22349170 10.1038/nrgastro.2012.14

[50] Feldman BV, Zinkl JG, Jain NC (2006) Schalm's Veterinary Hematology. Oxford, UK: Blackwell Publishing. 1344 p.

[51] Egeli AK, Framstad T, Morberg H (1998) Clinical biochemistry, haematology and body weight in piglets. Acta Vet Scand 39: 381–393. 9787501 10.1186/BF03547786 PMC8050675

[52] Trabalza-Marinucci M, Brandi G, Rondini C, Avellini L, Giammarini C, et al (2008) A three-year longitudinal study on the effects of a diet containing genetically modified Bt176 maize on the health status and performance of sheep. Livest Sci 113: 178–190.

[53] Adel-Patient K, Guimaraes VD, Paris A, Drumare M-F, Ah-Leung S, et al (2011) Immunological and metabolomic impacts of administration of Cry1Ab protein and MON 810 maize in mouse. PLoS ONE 6: e16346. 21298004 10.1371/journal.pone.0016346 PMC3029317

[54] Takeshita H, Mogi K, Yasuda T, Nakajima T, Nakashima Y, et al (2000) Mammalian deoxyribonucleases I are classified into three types: pancreas, parotid, and pancreas-parotid (mixed), based on differences in their tissue concentrations. Biochem Biophys Res Commun 269: 481–484. 10708579 10.1006/bbrc.2000.2300

[55] Einspanier R, Klotz A, Kraft J, Aulrich K, Poser R, et al (2001) The fate of forage plant DNA in farm animals: a collaborative case-study investigating cattle and chicken fed recombinant plant material. Eur Food Res Technol 212: 129–134.

[56] Mazza R, Soave M, Morlacchini M, Piva G, Marocco A (2005) Assessing the transfer of genetically modified DNA from feed to animal tissues. Transgenic Res 14: 775–784. 16245168 10.1007/s11248-005-0009-5

[57] Patterson JK, Lei XG, Miller DD (2008) The pig as an experimental model for elucidating the mechanisms governing dietary influence on mineral absorption. Exp Biol Med 233: 651–664. 10.3181/0709-MR-262 18408137

[58] Lehrer SB, McClain S (2009) Utility of animal models for predicting human allergenicity. Regul Toxicol Pharmacol 54: S46–S51. 19186207 10.1016/j.yrtph.2009.01.001

[59] Bailey M (2009) The mucosal immune system: Recent developments and future directions in the pig. Dev Comp Immunol 33: 375–383. 18760299 10.1016/j.dci.2008.07.003

[60] Rothkötter H-J (2009) Anatomical particularities of the porcine immune system - A physician's view. Dev Comp Immunol 33: 267–272. 18775744 10.1016/j.dci.2008.06.016

